# Structural ensemble-based docking simulation and biophysical studies discovered new inhibitors of Hsp90 N-terminal domain

**DOI:** 10.1038/s41598-017-18332-8

**Published:** 2018-01-10

**Authors:** Hyun-Hwi Kim, Ja-Shil Hyun, Joonhyeok Choi, Kwang-Eun Choi, Jun-Goo Jee, Sung Jean Park

**Affiliations:** 1 College of Pharmacy and Gachon Institute of Pharmaceutical Sciences, Gachon University, Incheon, 21936 Republic of Korea; 2 Research Institute of Pharmaceutical Researches, College of Pharmacy, Kyungpook, National University, Daegu, 41566 Republic of Korea

## Abstract

Heat shock protein 90 (Hsp90) is one of the most abundant cellular proteins and plays a substantial role in the folding of client proteins. The inhibition of Hsp90 has been regarded as an attractive therapeutic strategy for treating cancer because many oncogenic kinases are Hsp90 client proteins. In this study, we report new inhibitors that directly bind to N-terminal ATP-binding pocket of Hsp90. Optimized structure-based virtual screening predicted candidate molecules, which was followed by confirmation using biophysical and cell-based assays. Among the reported crystal structures, we chose the two structures that show the most favourable early enrichments of true-positives in the receiver operating characteristic curve. Four molecules showed significant changes in the signals of 2D [^1^H, ^15^N] correlation NMR spectroscopy. Differential scanning calorimetry analysis supported the results indicating direct binding. Quantified dissociation constant values of the molecules, determined by a series of 2D NMR experiments, lie in the range of 0.1–33 μM. Growth inhibition assay with breast and lung cancer cells confirmed the cellular activities of the molecules. Cheminformatics revealed that the molecules share limited chemical similarities with known inhibitors. Molecular dynamics simulations detailed the putative binding modes of the inhibitors.

## Introduction

Heat shock protein 90 (Hsp90) is one of the most abundant cellular proteins and accounts for 1–2% of the total amount of cytosolic protein. The main role of Hsp90 is to help the folding of client proteins as a chaperone. More than 200 proteins have been reported to be clients of Hsp90^1–3^. Consequently, through dynamic interactions with the client proteins, Hsp90 participates in a wide range of cellular processes including protein assembly, trafficking, folding, and degradation. The regulation of cellular processes by Hsp90 is more striking under stressed conditions such as high temperature and cancer, where the amount of Hsp90 increases by three-fold compared with that in normal circumstances. Hsp90 consists of three structurally distinct domains: N-terminal (Hsp90N), middle (Hsp90M), and C-terminal domains (Hsp90C). Hsp90 exists as a dimer via intermolecular contacts between the Hsp90Cs. Hsp90M is thought to provide the main binding sites for client proteins. ATP binding to Hsp90N triggers a conformational change from an open state in which the two Hsp90Ns are separated to a closed state in which the Hsp90Ns are dimerized. The less characterized Hsp90C also possesses a site for nucleotide binding, seemingly associated with allosteric regulation of Hsp90^4^.

Hsp90 interacts with about 60% of all kinases in human^5^. The participation of cochaperone Cdc37 is indispensable for interactions with kinases. Inhibition of Hsp90 results in ubiquitination-mediated degradation of the client kinases. The confirmation of the roles of these interactions in tumour cells has brought about the constant interest in developing anticancer agents targeting Hsp90^6–11^. Most of the potential chemical inhibitors have focused on the ATP-binding site of Hsp90N. The structure of the complex with geldanamycin shows how the inhibitors causes the dissociation of the client kinase^12^. Recent studies have also reported molecules that can inhibit Hsp90 through binding to the C-domain^13^. While no inhibitor of Hsp90 is currently approved as a drug, several molecules are being tested in late clinical trial stages. In addition to kinases, Hsp90 can interact with other disease-related proteins such as p53 and tau, also assisting with protein folding^14–17^. However, the roles of Hsp90 and potential therapeutic strategies through controlling Hsp90 function have remained elusive in diseases other than cancer.

Structure-based virtual screening (SBVS) has played a complementary role in combination with high-throughput screening for discovering hit compounds in the early stages of the drug discovery process^18,19^. Despite the technical advances and successes, however, SBVS suffers from a high false-positive rate in the selected candidates. Even compounds that perfectly fit into the target site of a protein with *in silico* calculations can scarcely bind to the protein. Two central causes for this unsatisfactory performance are the conformational changes in the receptor upon binding to a ligand and the case-dependent performances of docking algorithms in prioritizing true-positives. Hsp90N has been regarded one of the most difficult targets for applying SBVS as reflected by the relatively poor enrichments of true-positives in DUD-E docking benchmarks^20^. In this study, we have implemented an ensemble-based virtual screening to improve the performance of SBVS. First, a test of the reproduction of the poses of the inhibitors found in X-ray structures enabled the selection of the most suitable software. Second, the best structures were chosen with known inhibitors and their physicochemically matched decoys using the algorithm. Third, high-throughput SBVS (HTSBVS) was carried out using the pairs of software and structures. Fourth, 2D NMR was used to confirm the hits and quantify the binding affinities. Fifth, cell-based experiments confirmed the inhibitory activities of the four newly discovered inhibitors. Finally, computational studies, including cheminformatics and molecular dynamics (MD) simulation, were used to help elucidate the detailed features and roles of the identified moieties.

## Results

### Selection of algorithm for high-throughput structure-based virtual screening

**Figure 1:**
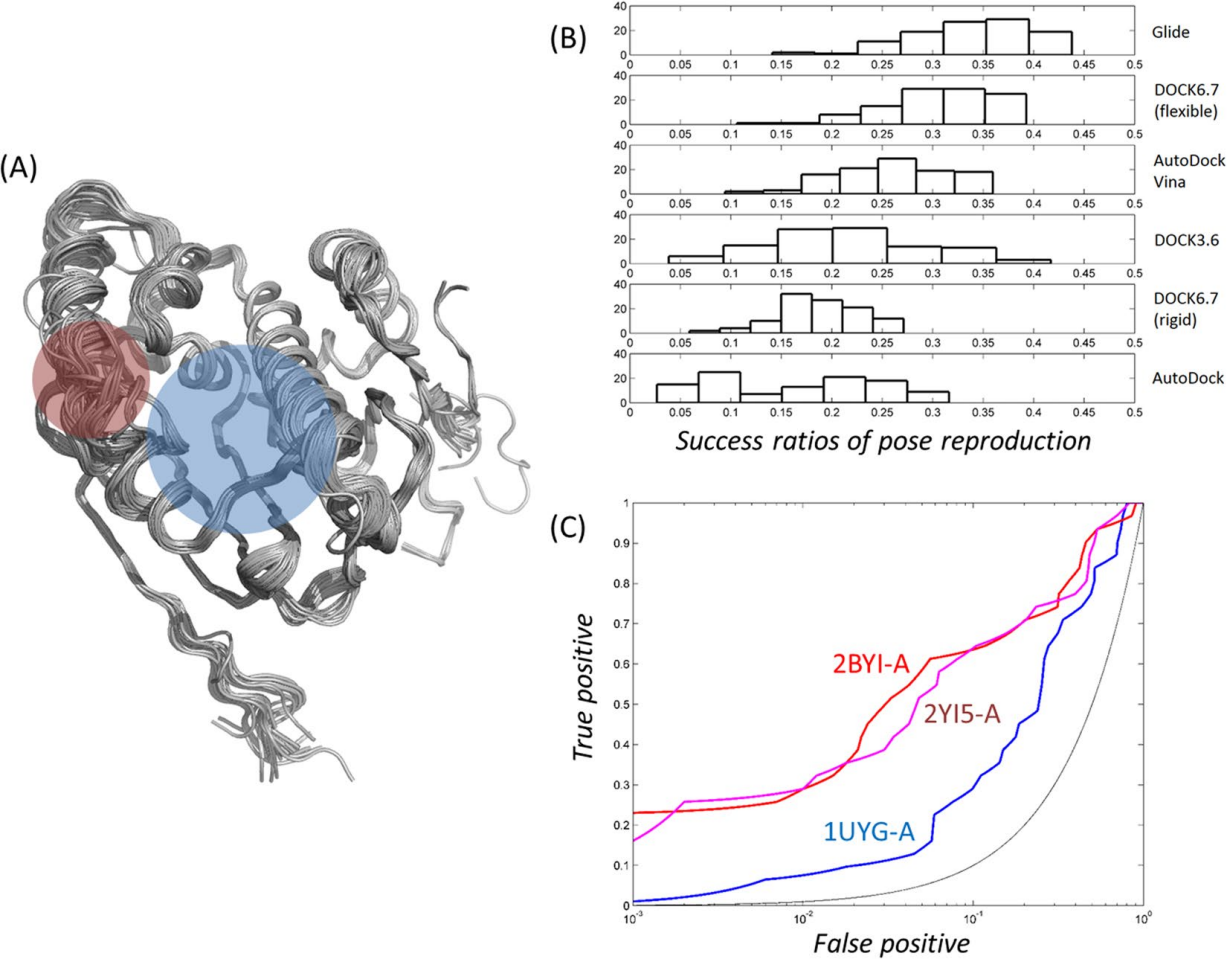
Structural ensemble, distributions of pose reproductions by algorithms, and structure-dependent profiles of ROC curves. (**A**) The ensemble of 108 crystal structures is represented with the substrate-binding pocket coloured in blue and the position of Leu-107 in red. (**B**) Algorithm-dependent success rates in reproducing the poses of the inhibitors found in X-ray structures are represented as histograms. The 90 crystal inhibitors were docked into the structures of Hsp90N with 6 algorithms from 5 software programs. A docked pose is judged as a success when it overlapped with that of the crystal structure within 2 Å in the heteroatoms. (**C**) Receiver operating characteristic (ROC) curves from two structures for high-throughput virtual screening in this study are drawn in red (2BYI-A^28^) and magenta (2YI5-A^29^). The blue line corresponds to that of 1UYG-A^51^, which was used as the DUD-E benchmark^20^. Random enrichment is shown in black for comparison. In the ROC curves, the x-axis is logarithmically scaled to emphasize the earlier enrichments of true-positives. The values of LogAUC for 2BYI-A, 2YI5-A, and 1UYG-A are 33.5, 31.9, and 12.9, respectively.

The poses and energies of the docked chemicals reflect the performance of the HTSBVS. Poses predicted by SBVS should be similar to those found in crystal structures. True-positives should have lower energies than false-positives, enabling the earlier enrichments of true-positives in HTSBVS. The combined use of the best pair of software and structure can provide improvements in poses and energies. We first selected the best algorithm by testing which software could most faithfully reproduce the poses of inhibitors found in X-ray crystal structures (Fig. 1). Inhibitors with crystal structures available in complex states were docked into the Hsp90N structures using six algorithms from five software programs [AutoDock^21^, AutoDock Vina^22^, DOCK 3.6^23^, DOCK 6.7^24^ (anchor-and-grow and rigid methods), and Glide-SP^25,26^]. The docked poses in each structure were compared with those found in crystal structures. The predicted poses that could be overlapped with the experimental ones within 2 Å of the root mean square deviation (RMSD) were judged as successful. Glide showed an average success rate of 33.9%, followed by DOCK 6.7 (anchor-and-grow, 30.3%), AutoDock Vina (26.1%), DOCK 3.6 (21.4%), DOCK 6.7 (rigid, 18.7%), and AutoDock (16.6%) (Fig. 1). The highest success rates for Glide, DOCK 6.7 (anchor-and-grow and rigid), DOCK 3.6, AutoDock, and AutoDock Vina were 47.0, 42.4 and 29.4, 45.8, 34.7, and 38.8%, respectively. Therefore, we selected Glide as an engine for subsequent dockings.

### Selection of template structure and high-throughput structure-based virtual screening

We then chose the best structure by performing SBVS in the crystal structures with 33 inhibitors extracted from the BindingDB^27^ (Fig. S1) and 2370 of their property-matched but topologically different decoys (Fig. 1). Here, the inhibitors were filtered to be similar with a Tanimoto coefficient (Tc) value less than 0.6. The performance was judged based on logarithmically scaled area under the curve (LogAUC) based on receiver operating characteristic curve (ROC). A higher value of LogAUC reflects the early enrichment of the true-positives more faithfully than using the AUC value. Considering faithful pose reproduction and LogAUC, two structures, 2BYI-A (Chain A from Hsp90N with PDB ID of 2BYI)^28^ and 2YI5-A^29^, were selected. In 2BYI-A and 2YI5-A, 38% and 39%, respectively, of the reproduced poses were judged as successes. The values of AUC, LogAUC, and enrichment at 1% (EF1) were 83.5, 33.5, and 25.9, respectively, in 2BYI-A and 83.0, 31.9, and 27.62, respectively, in 2YI5-A. Figure 1 shows the ROC curves of 2BYI-A and 2YI5-A. It is noteworthy that the averaged values of AUC, LogAUC, and EF1 for all 108 crystal structures are 75.7, 21.0, and 16.3, respectively. The backbone RMSD between 2BYI-A and 2YI5-A is 1.08 Å. Despite the close similarity in the structures, however, no top candidates in the HTSVS were overlapping between the two, again demonstrating the heavy dependence of HTSBVS on the template structure in use. We purchased the top 30 compounds from the results of both 2BYI-A and 2YI5-A, comprising 60 in total, based on the Glide-SP scoring functions (Fig. S2).

### Two-dimensional NMR experiments identified direct binders

**Figure 2:**
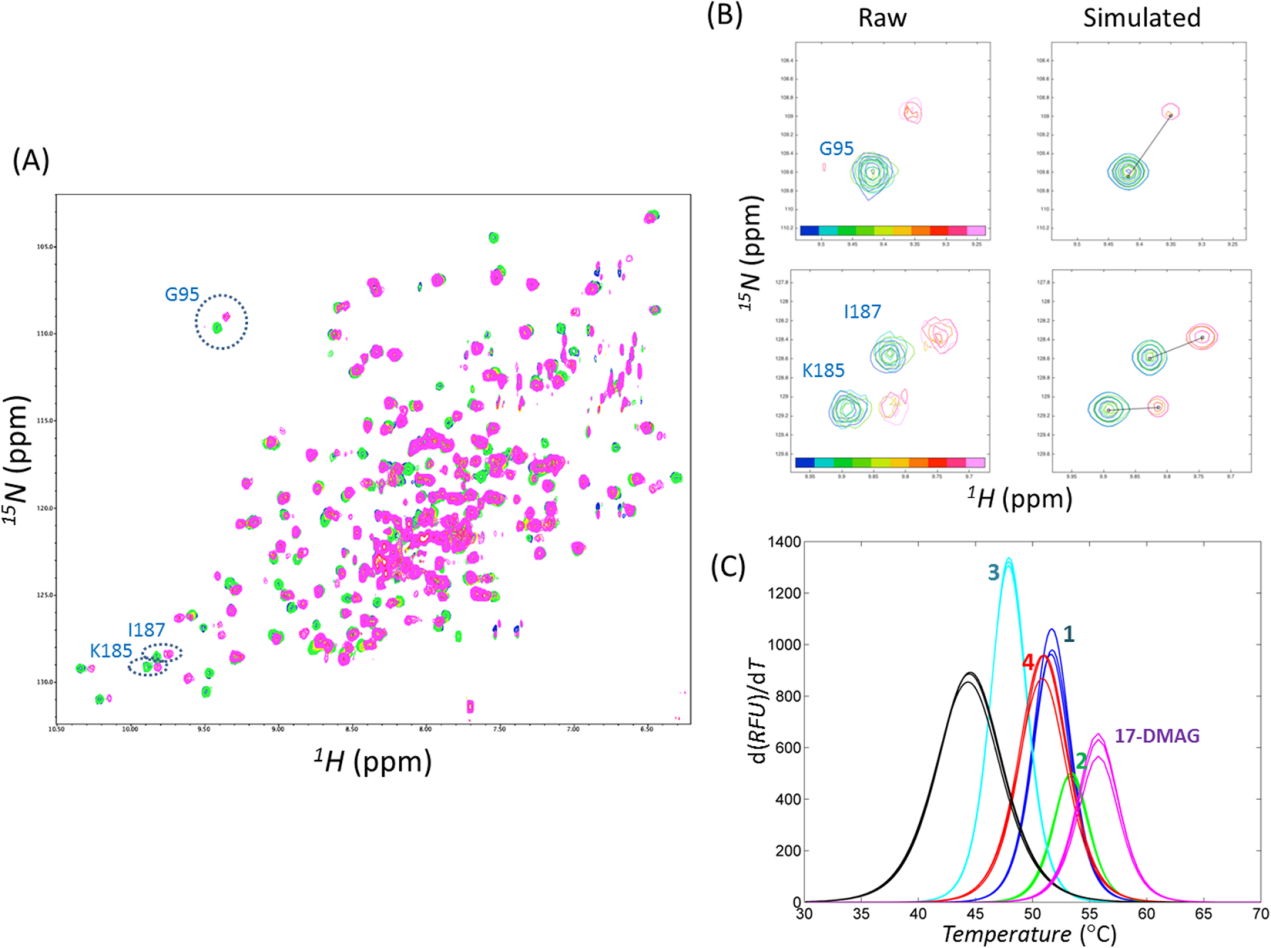
Quantification of inhibitor binding using 2D NMR and differential scanning fluorimetry. (**A**) Example of 2D [^1^H, ^15^N] HSQC in the titrations with **1**. A series of ligands with different concentrations in 1:0 to 1:2 protein:ligand ratios were added. NMR peaks of 1:0 and 1:2 ratios are coloured in green and magenta, respectively. The residues exemplified in calculating K_d_ are circled. (**B**) The simulated NMR peaks by line-shape analyses for calculating K_d_ of **1** are drawn for comparison with the raw data. (**C**) Differential scanning fluorimetry in the apo and holo states are represented. Differentials of relative fluorescence units (RFU) for temperature are plotted in blue, green, cyan, red, magenta colours for **1**–**4** and 17-DMAG, respectively. Black lines correspond to the profiles of apo states. Calculated differences of mid-point melting temperatures from that of apo state (44.4 °C), ΔT_m_s, in **1**–**4** and 17-DMAG are 7.3, 8.9, 3.5, 6.5, and 11.3 degrees, respectively.

**Figure 3:**
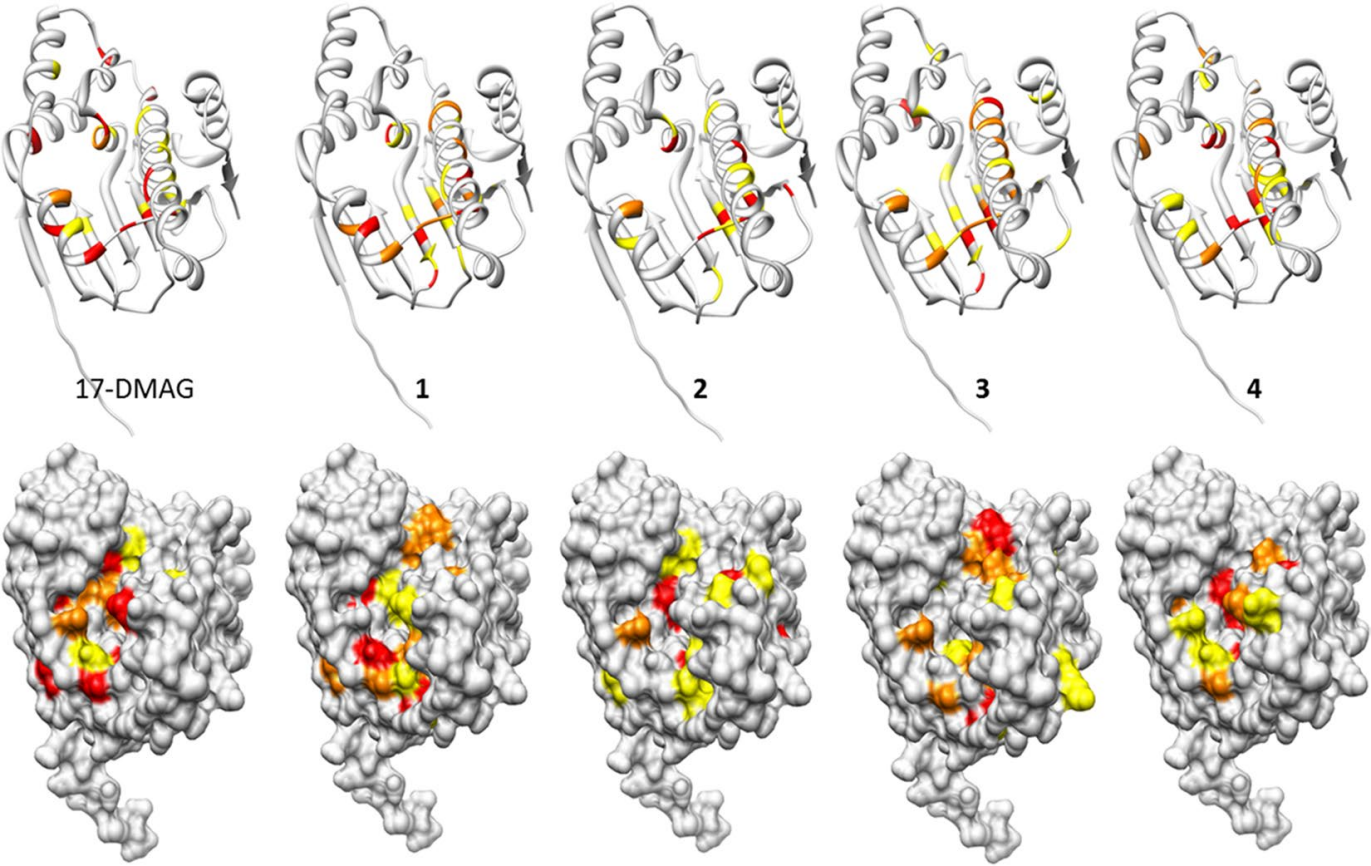
Mapping of perturbed residues by 2D NMR onto 3D structure. The changes of 2D [^1^H, ^15^N] HSQC in the titrations are quantified as ΔCS using $\sqrt{{0.5\times ({\rm{\Delta }}{\rm{H}}}^{2}+{(0.2\times {\rm{\Delta }}{\rm{N}})}^{2})}$. Here ΔH and ΔN mean the chemical shift difference between apo and holo forms in ^1^H and ^15^N dimensions. Once calculating the mean and standard deviation (SD) values of ΔCS in the cases of ΔCS > 0, the residues of Hsp90N are classified into four criteria: (i) ΔCS ≤ mean + SD, (ii) mean + SD < ΔCS ≤ mean + 2 SD, (iii) ΔCS > mean + 2 SD, and (iv) disappeared. The residues coloured in yellow, red, and orange correspond to the cases of (ii), (iii), and (iv), respectively.

Of the biophysical methods for studying protein-ligand interaction, 2D [^1^H, ^15^N] correlation NMR spectroscopy is the method that generates fewer false-positives^30,31^. Compared with other methods that generate an averaged, single, and overall pattern, all the peaks in 2D NMR can probe intermolecular interaction. The existence of specific changes in 2D NMR peaks upon the addition of a small molecule may be sufficient to judge the molecule as a real binder. We observed noticeable signal changes when adding compounds **1** (ZINC09350001), **2** (ZINC00302593), **3** (ZINC04643798), and **4** (ZINC04757705) (Table 1). Interestingly, they share some apparent similarities with each other. **1**–**3** contain resorcinol moieties linked with pyrazole; on the contrary, **4** has triazole in the position corresponding to pyrazole in **1**–**3**. The perturbed patterns of 2D NMR mimicked those of the reference, 17-DMAG (Figs 2 and S3), though the quantified changes differed with **1**–**4**. The mapping of the perturbed residues onto 3D structure allowed the confirmation that **1**–**4** bind to ATP binding site of Hsp90N (Figs 3 and S4). Because the perturbations happened at the slow exchange, we employed line-shape analyses to calculate K_d_ values using a series of 2D [^1^H, ^15^N] HSQC with different ligand concentrations^32^. The calculated K_d_ values for **1**–**4** were 0.35, 0.10, 33.1, and 5.0 μM, respectively. On the other hand, the K_d_ value of 17-DMAG was fitted as 20 nM. Please note that the reported IC_50_ value of 17-DMAG with fluorescence polarization was 62 nM^33^. We also measured the change in the T_m_ value using differential scanning fluorimetry (DSF) to support direct binding (Fig. 2C). Apo state showed the T_m_ value of 44.4 °C. Remarkably, the order of the elevated T_m_ values in **1**–**4** and 17-DMAG of 7.3, 8.9, 3.5, 6.5, and 11.3, respectively, are entirely consistent with the 2D NMR data, suggesting that direct binding led to the stabilization of Hsp90N.

**Table 1:** Details of the compounds identified as new inhibitors of the Hsp90 N-terminal domain.

**ID**	**ZINC ID**	**2D structure**	**MW**	**LogP** ^**&**^	**K**_**d**_ **(μM)**	**LE** ^**#**^
**1**	ZINC09350001		315	3.79	0.35	0.40
**2**	ZINC00302593		300	3.73	0.10	0.44
**3**	ZINC04643798		284	3.60	33	0.29
**4**	ZINC04757705		405	2.98	5	0.30

^&^Is the registered value in the ZINC database^69,70^.

^#^Is calculated as −1.37 × (Log_10_K_d_)/HA, where HA is the number of heteroatoms^42^.

### Cell-based activity test confirmed the cellular activities of the inhibitors

**Figure 4:**
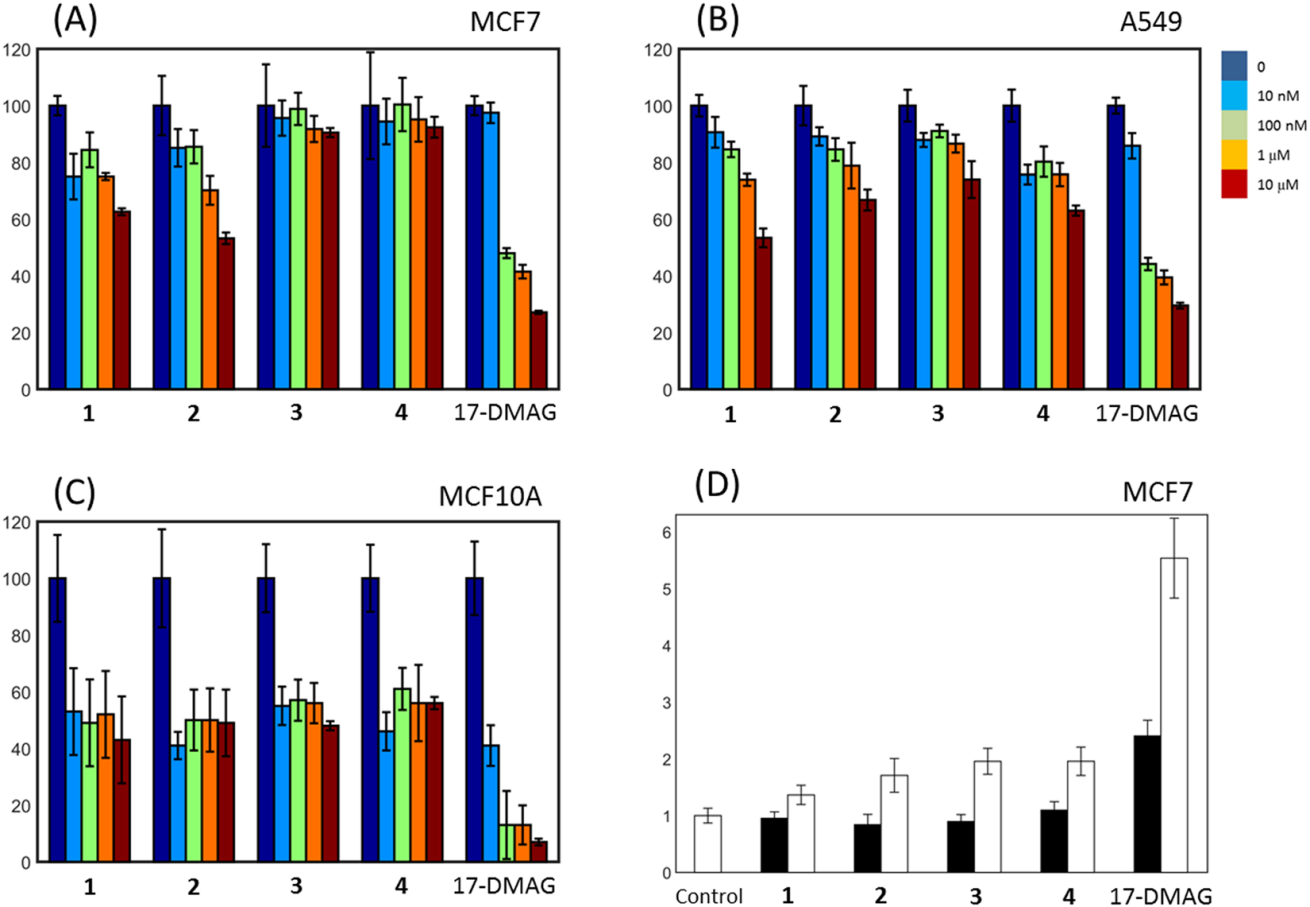
Cell growth inhibition by **1**–**4**. Cell growth inhibitions were observed in (**A**) MCF7, (**B**) A549, and (**C**) MCF10A cells. Three experiments were repeated in a condition to generate standard deviations that are represented by the error bars. 17-DMAG was used as a positive control. (**D**) The amounts of Hsp70 mRNA were quantified using real-time PCR in MCF7 cells. The filled and open bars indicate the measurements in 3 and 6 h, respectively. The concentrations of the added **1**–**4** were 10 μM, whereas that of 17-DMAG was 100 nM.

Whereas only **1** and **2** were significantly inhibitory to MCF7 cells (Fig. 4A), all four compounds inhibited the growth of A549 cells in a concentration-dependent manner (Fig. 4B). The decreased growths using all concentrations were comparable to or smaller than the decreased growth caused by 17-DMAG for both MCF7 and A549 cells. To know the effects of **1**–**4** in non-transformed cells, we tested the cell growth inhibition in MCF10A cells that originate from human breast mammary gland as well. The addition of the inhibitors resulted in the cell growth inhibition, but no explicit concentration dependency was observed for **1**–**4** within uncertainty (Fig. 4C). The hallmark response of Hsp90 inhibition is the induction of Hsp70. We further checked mRNA levels in MCF7 cells using RT-PCR upon the addition of **1**–**4** and 17-DMAG in 3 and 6 h. Meaningful time-dependent increments were detected (Fig. 4D). These data will suggest that **1**–**4** have activity through Hsp90 in the cellular levels.

### Cheminformatics retrieved the chemical novelties and chemicals similar to the hit molecules

**Figure 5:**
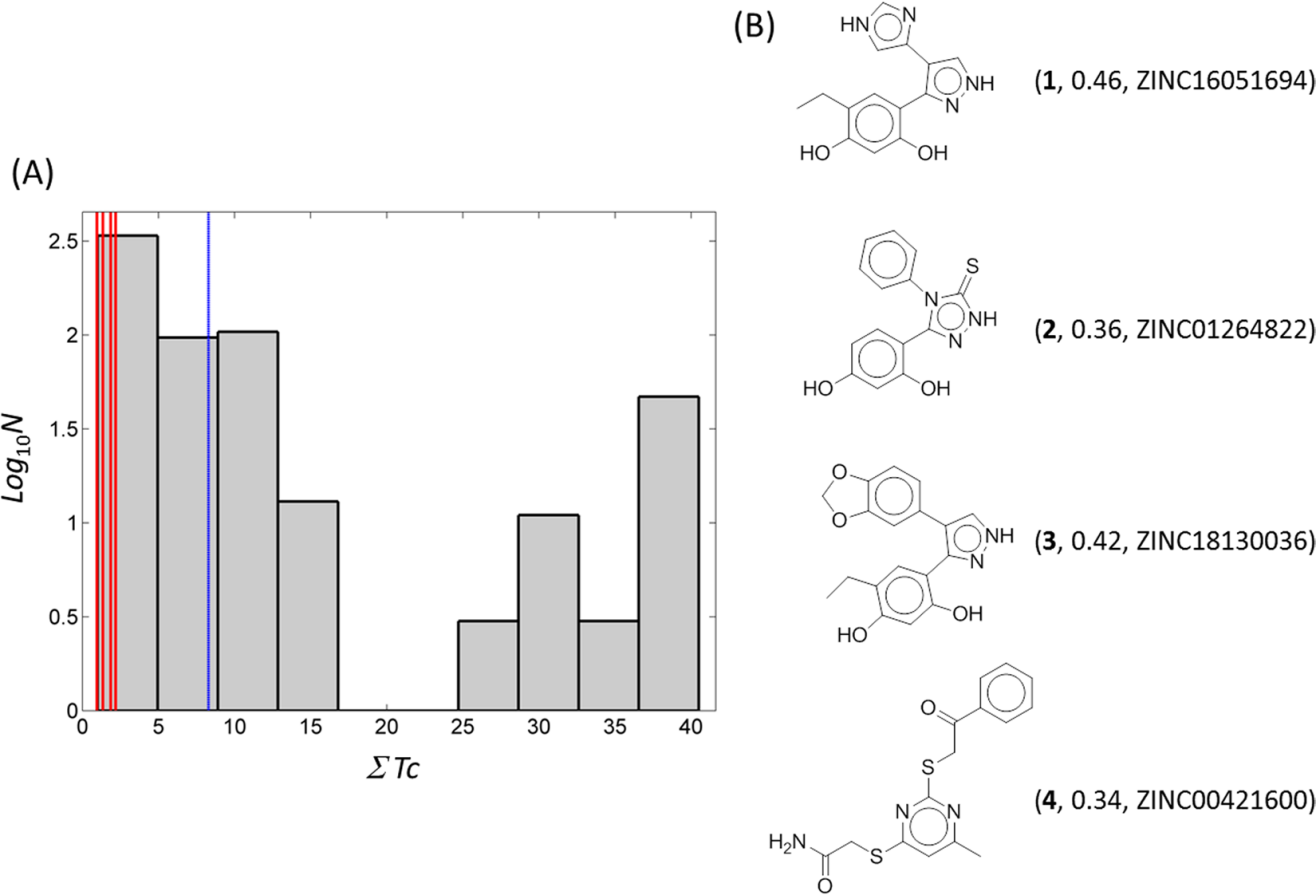
Cheminformatics for **1**–**4**. Similarity ensemble approach was employed to quantify the chemical similarities of **1**–**4** and known Hsp90N inhibitors. Tanimoto coefficients (Tcs) between a molecule and 617 BindingDB-deposited inhibitors^27^ were summed to form an individual ∑Tc. (**A**) The ∑Tc of the 617 known inhibitors represented using a histogram as reference. The mean value of the ∑Tc distribution, 8.28, is drawn as a blue line. The ∑Tc for **1**–**4** was also calculated and is represented as red lines. The ∑Tc for **1**–**4** were 1.86, 1.35, 2.19, and 0.96, respectively. Only Tcs greater than the threshold, 0.3, were considered. (**B**) The chemicals most closely related to **1**–**4** by Tc from the BindingDB-deposited Hsp90N inhibitors are shown. The corresponding hit compound, Tc, and ZINC IDs are given in parentheses.

We then searched for the closest known inhibitors of **1**–**4** in terms of chemical similarity by Tc from BindingDB, which contains Hsp90N inhibitors confirmed directly by the enzyme-based experiments. Corresponding molecules were ZINC16051694 (Tc = 0.46, K_d_ = 280 nM)^34^, ZINC01264822 (0.36, IC_50_ = 0.5 nM)^35^, ZINC18130036 (0.42, IC_50_ = 2 μM), and ZINC00421600 (0.34, IC_50_ = 50 μM) for **1**–**4**, respectively (Fig. 5). The data with ZINC18130036 and ZINC00421600 came from the PubChem BioAssay database (AID: 712)^36^. In order to compare the chemical similarity in detail, we employed the similarity ensemble approach (SEA)^37^. In SEA, the pairwise Tc values between two molecules are summed to form ΣTc. By comparing the ΣTc in a test molecule and the distribution of ΣTc in a set of small molecules, we can compare the chemical novelty of a test molecule. In this study, only Tc values greater than 0.3 were considered for clarity. A higher value of ΣTc indicates that a molecule shares more chemical similarity to the others. The values of ΣTc were 1.86, 1.35, 2.19, and 0.96 for **1**–**4**, respectively, which correspond to 453rd, 515th, 425th, and 617th of 617 Hsp90N inhibitors. The mean value of ΣTc was 8.28 in the known inhibitors, with 41.57 as the largest value for a macrocyclic o-aminobenzamide, ZINC71340643^38^. Visualization of the ΣTc distribution clearly demonstrates the limited chemical similarities of **1**–**4** with the other inhibitors (Fig. 5). On the other hand, the pairwise Tc values in **1** & **2**, **1** & **3**, **1** & **4**, **2** & **3**, **2** & **4**, and **3** & **4** are 0.25, 0.35, 0.19, 0.38, 0.18, and 0.15, respectively. We next searched for known bioactivities of **1**–**4** in the ChEMBL database^39^. There was no known bioactivity for **1**–**4**.

### Docking and MD simulations proposed the binding poses

**Figure 6:**
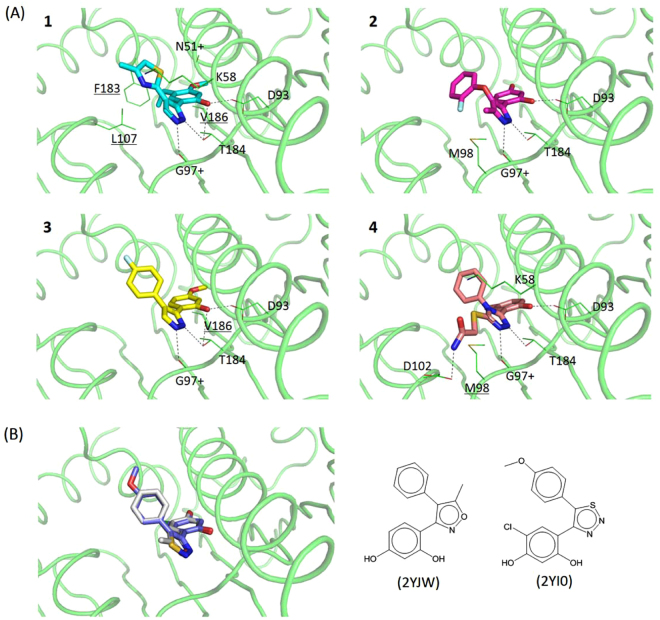
Simulated poses of **1**–**4** in complex with Hsp90N. (**A**) **1**–**4** were docked into 2BYI-A. The hydrophilic interactions are shown with dashed lines. The underlined residues are involved in hydrophobic interactions, whereas the others are involved in hydrophilic interactions. The residues with “+” indicate those that interact with backbone atoms. The structures of Hsp90N are the same direction as that in Fig. 1. (**B**) Ligands similar to **1**–**4** in the Hsp90N complex structures, 2YJW^40^ and 2YI0^29^, are overlaid based on their protein coordinates. The white and purple sticks correspond to 2YJW and 2YI0, respectively. The Tc value between 2YJW and **2** is 0.36, while that between 2YI0 and **3** is 0.34.

**Figure 7:**
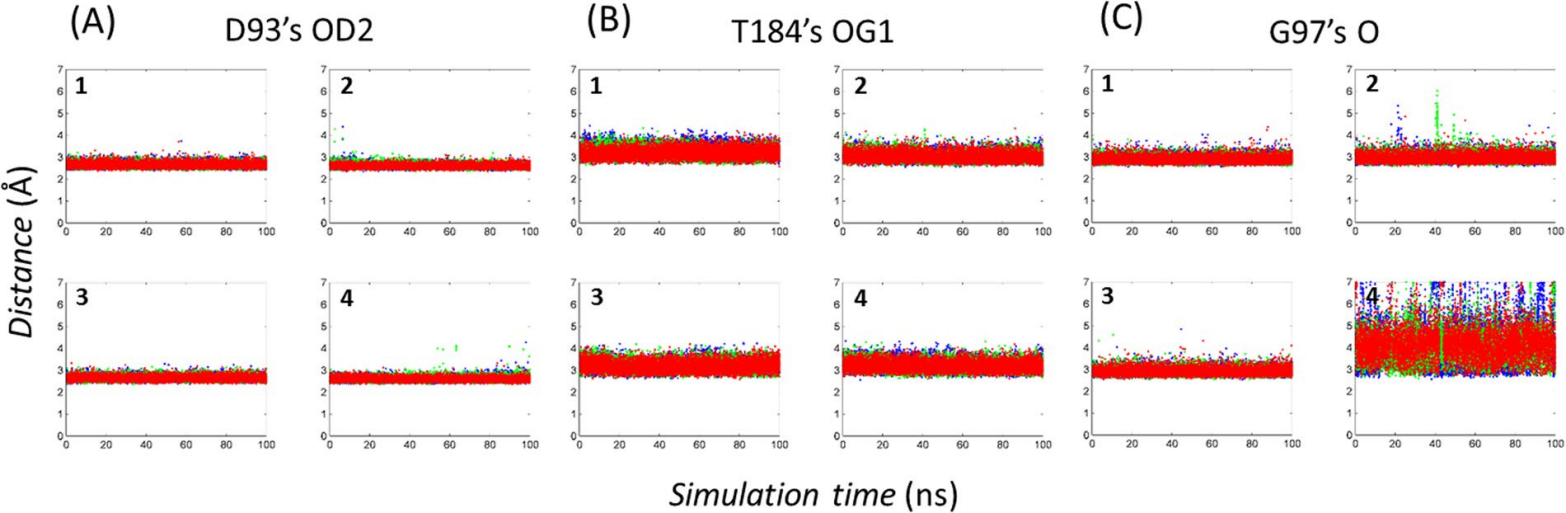
Molecular dynamics simulations of complex structures with **1**–**4**. The time-dependent distances between the heteroatoms forming intermolecular hydrogen bonds are drawn from 100 ns molecular dynamics (MD) simulations. Each MD simulation of a complex with **1**–**4** was repeated three times with different random seeds, coloured in blue, green, and red. Asp-93′s OD2 (**A**) and Gly-97′s O (**C**) were used as hydrogen bond acceptors, whereas Thr-184′s OG1 (**B**) was a donor.

Figure 6A depicts the binding modes of **1**–**4** with docking simulations using 2BYI-A. The predicted positions are entirely overlapped with those for ATP binding, as reflected by 2D NMR. Remarkably, the key interactions in the models are conserved in **1**–**4** although the pairwise topological similarities between **1** and **4** are limited. All four have intermolecular hydrogen bonds with the OD2 of Asp-93, the OG1 of Thr-184, and the O of Gly-97. An additional hydrogen bond with Asp-102 existed in **4**. **1** and **4** included electrostatic interactions through the side chain of Lys-58. There were hydrophobic contacts through Leu-107, Phe-183, and Val-186 in **1**. **3** and **4** revealed hydrophobic interactions by Val-186 and Met-98, respectively. Met-98 is involved in an electrostatic interaction in **2**. These interactions are common in other known inhibitors. Comparison of the binding modes of the 90 known inhibitors found in X-ray structures indicated that 66, 22, and 21 inhibitors contact the side chains of Asp-93, Thr-184, and Lys-58, respectively. In particular, the hydrogen bonds through Asp-93 and Thr-184 are found in the crystal ligands of acyclic radicicol analogues. For instance, ligands with limited similarities to **2** (PDB ID: 2YJW, Tc = 0.36) and **3** (2YI0, 0.34) have identical hydrogen bonds^29,40^, supporting the reliability of the docked models (Fig. 6B). MD simulation assessed the predicted binding modes in detail (Fig. 7). The overall folds of Hsp90N in complex with inhibitors showed no significant changes compared with the starting structure. The residence time of a contact in an MD simulation can qualitatively reflect the importance of the interaction. Of the intermolecular hydrogen bonds, those through the OD2 of Asp-93 remained almost invariable. In **1**–**4**, the occupancy ratios were more than 99% (Fig. 7A). The hydrogen bonds through the OG1 of Thr-184 were found with the respective occupancies of 15, 48, 20, and 14% in **1**–**4** (Fig. 7B). The hydrogen bonds by the O of Gly-97 existed with residential portions of 82, 66, and 78% for **1–3**, respectively. On the contrary, the O of Gly-97 in **4** was not used for the intermolecular hydrogen bond, occupying 5% of the trajectories (Fig. 7C). Similarly, the hydrogen bond through the OD1 of Asp-102 in **4** was unstable appearing at about 12%. The results indicate that the detailed features of the intermolecular hydrogen bonds differ between **1–3** and **4**, although the docked models share apparent similarities. The difference may be caused by the improper location of water molecules that play substantial roles in the intermolecular interactions between Hsp90N and inhibitors^6^.

## Discussion

The main purpose of SBVS is to find new chemotypes that are difficult to identify by simple quantitative structure–activity relationships. Nevertheless, hits with a new chemotype having higher activity will be preferred in the optimization process for lead compounds. Because the quantified inhibitions of hits by SBVS are dependent on the physicochemical features of the target sites as well as the assays employed, it would be inconclusive to directly compare the data. Even considering these caveats, the sub μM inhibitions observed in **1** and **2** are somewhat stronger than those commonly measured for hits identified by SBVS^41^. In particular, the ligand efficiency metrics for **1** (0.40) and **2** (0.44) show promise for further optimization^42^. Several papers have reported new hits against Hsp90N using SBVS^43–46^. One of them employed 2D NMR to quantify the inhibition of three hits with K_d_ values in the range of 2 to 20 μM^43^. Our 2D NMR screen used 1:1 ratios of protein and ligand. This condition is more suited to detect strong inhibitors because the population of the protein in complex varies roughly depending on the K_d_ in the system. Indeed, the change of the chemical shifts in **1**–**4** was the largest in **2** followed by **1**. On the contrary, if the ligand is more concentrated than the protein (>10-fold), the portion of the bound form relies less on the K_d_, and has the advantage of detecting weak binders. Two-dimensional NMR is a robust method to minimize the identification of false-positives such as Pan-Assay INterference compoundS (PAINS)^30,47^ as hits. The four hits in this study scarcely share chemical similarity to the known PAINS^48^. We additionally employed DSF as an orthogonal method to 2D NMR to confirm the direct bindings. It is noteworthy that Hsp90N alone has little ATP catalyzing activity^49^. Therefore the quantified confirmation of the direct binding between Hsp90N and a small molecule is important to interpret the accompanying cellular activities. Our approach could be applied for discovering the small molecules that modulate the protein-protein interaction in a similar way. Here we would like to stress that the careful 2D NMR-based assays became practically applicable since SBVS had reduced the number of test samples.

**Figure 8:**
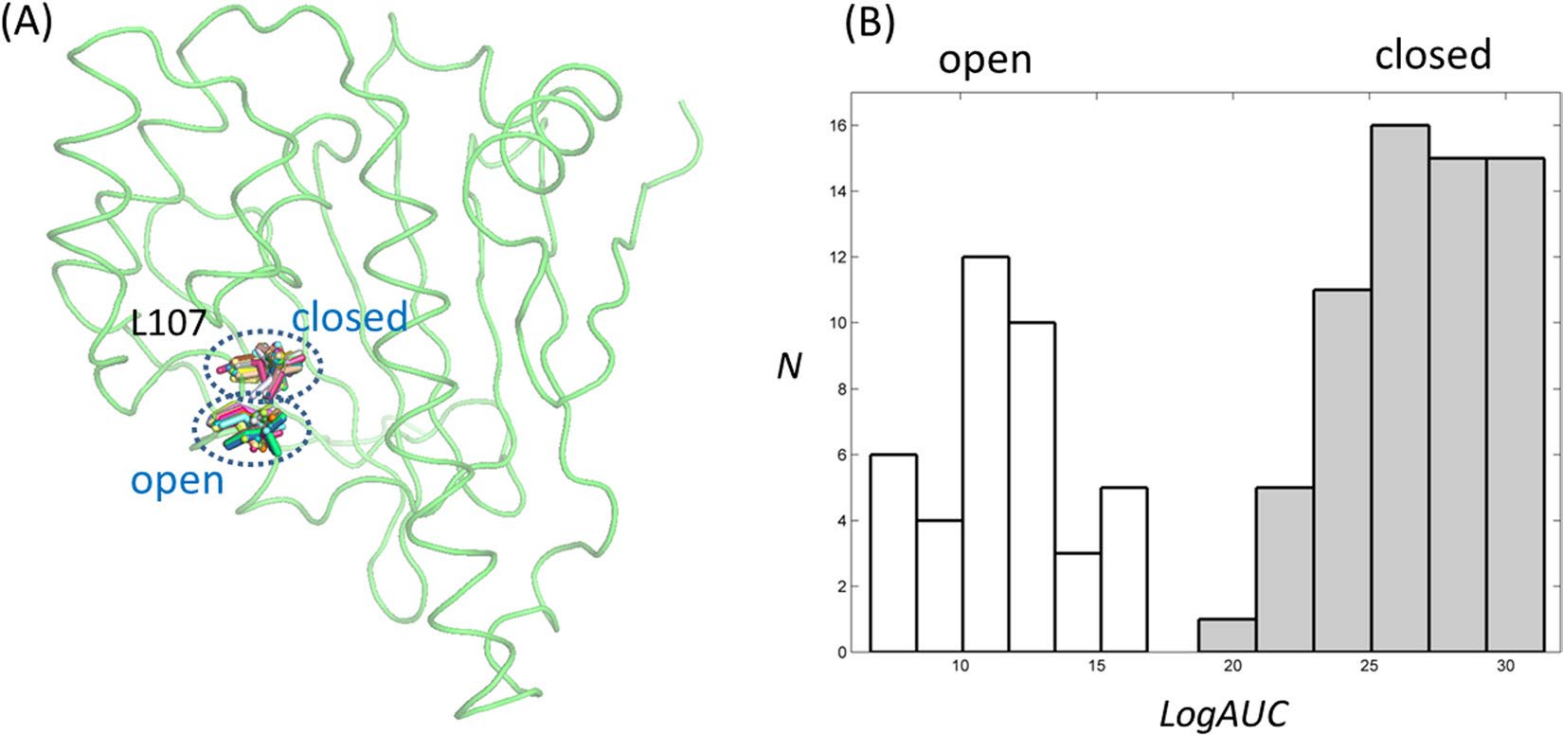
Classification of Hsp90N structures based on Leu-107. (**A**) Crystal structures of Hsp90N were classified based on the position of Leu-107. All structures are rotated from that shown in Fig. 1 by 60 degrees along the x-axis for clarity. Two groups are labelled as open and closed according to the Leu-107 position. (**B**) The distribution of LogAUCs in the structures of the two groups. Those from the open forms are drawn in white, whereas the closed forms are represented in grey.

Hsp90N is known as a problematic case for enriching true-positives using SBVS. In the DUD-E database^20^, the values for AUC, LogAUC, and EF1 for Hsp90N are 69.4, 14.6, and 3.4, which is much worse than the averaged values for 101 other cases in the DUD-E database (76.5, 24.4, and 19.8 for AUC, LogAUC, and EF1, respectively). The current results using the selected pairs of docking software and structures (83.5/33.5/25.9 with 2BYI-A and 83.0/31.9/27.6 with 2YI5-A) had improved metrics. Repasky *et al*. reported that the poor enrichment in targeting Hsp90N with SBVS stemmed from the flipped position of Leu-107 as the open form^50^. They showed that the exclusive selection of the closed form with new scoring could result in improved enrichment^50^. We investigated the features of experimentally determined Hsp90N structures. The crystal structures of Hsp90N can be divided into either closed or open forms. The closed form described approximately 62% (67/108) of the structure, while 38% (41/108) of the structures were the open form (Fig. 8). The conformations of Leu-107 in 2BYI-A and 2YI5-A are classified as the closed forms, whereas 1UYG-A that is used for DUD-E takes the open form^51^. Histogram analyses of the LogAUC values in the two cases clearly show the better enrichments in the closed form (Fig. 8). The averaged values of LogAUCs are 27.0 and 11.6 in the closed and open forms, respectively. To know the general applicability of our SBVS, we also tested another problematic case from DUD-E database^20^. MCR, mineralocorticoid receptor, is the case that showed the poorest enrichment of true-positives among the exemplified 102 cases. The metrics from ROC were 36.3 (AUC), −4.15 (LogAUC), and 2.1 (EF1). The use of 36 crystal structures of MCR and DOCK 3.6 successfully improved the metrics to 75.7 (AUC), 29.0 (LogAUC), and 34.8 (EF1) as shown in Fig. S5, supporting the generality of the approach.

Then whether could the open form structure of Hsp90N reproduce the poses of **1**–**4**? The docked poses of **1**–**4** were completely different each other in the cases of 2BYI-A and 1UYG-A (Fig. S6). Also, the docking with **1**–**4** and their property-matched 200 decoys showed that **1**–**4** were hardly retrieved with the lower scores in the open form, whereas **1**–**4** were ranked with the lowest scores in the closed form (Fig. S6). Interestingly, the numbers of van der Waals contacts between Leu-107 and **1**–**4** were much more in the open form than the closed form. It indicates that the location of the region around Leu-107 directly influences positioning of **1**–**4**. Please note that in this study we neither made any assumption on the conformation nor employed tailored scoring functions and instead selected the structure showing the best profile for HTSBVS. This may suggest the generality of our approach. The results are consistent with previous reports where the use of the structural ensemble increased the success rate of initial hits^52–54^. If the experimentally available structures are insufficient in number, an ensemble prepared by MD simulations would be an alternative^55^. In this study, we did not alter the algorithm once deciding the best software to use. The paired algorithm and structure can be searched simultaneously, although this requires substantial calculation times. This will be the subject of a future study.

The cell growth inhibitions of **1**–**4** and 17-DMAG seem qualitatively consistent with the *in vitro* binding affinities. It would imply that the inhibition of Hsp90N under the cellular environments occurs largely dependent on the strength of the direct binding. Meanwhile, one may question about the quantitatively less correlated activities in the protein-binding and cell-based assays. It is equivocal to link the binding affinities with the cellular activities, but one possible explanation would be the difference in the pharmaceutical features. Compared with 17-DMAG that has been tested in clinical stages after the extensive modifications by medicinal chemistry, **1**–**4** are hits in the current stage. The cell permeability and solubility under cellular environments as well as the efficacy of **1**–**4** are less optimized. Of the Hsp90N inhibitors in clinical development, the compounds chemically closest to **1**–**4** are AUY922^56^ and STA9090^57^. Both are classified as the resorcinol analogues from radicicol^11^. Radicicol, also known as monorden, is a macrolactone natural product from *Diheterospora chlamydosporia*. Since radicicol inhibits topoisomerase^58^, bacterial sensor kinase PhoQ^59^, and pyruvate dehydrogenase kinase^60^ by direct binding to the ATP binding pockets of these enzymes, there is a possibility that **1**–**4** inhibit the enzyme in a similar manner. These proteins are classified as the members of GHKL (Gyrase, Hsp90, Histidine Kinase, MutL) that share an evolutionarily conserved ATPase domain^61^. In fact, some resorcinol compounds inhibit both Hsp90N and pyruvate dehydrogenase kinase^62,63^. A related issue is the selective inhibition for the paralogue Hsp90N of Hsp90α, Hsp90β, Grp94, and TRAP1^64^. As reported by recent papers, it has become feasible to develop organelle-specific Hsp90N inhibitors^65^. Simple comparison indicates that the contacting residues in Hsp90N for **1**–**4**, Asp-93 and Thr-184, are all conserved in the four isoforms, increasing the possibility that the hits are inhibitory for the paralogs of Hsp90N. However, the actual inhibition and the detailed dynamic features such as the residence times of intermolecular hydrogen bonds remain to be addressed.

In conclusion, we have applied SBVS to find new inhibitors of Hsp90N, which has been known as a case with poor enrichment of true-positives by SBVS. Use of an ensemble of experimental structures has resulted in the finding of new types of inhibitors, **1**–**4**, coupled with 2D NMR experiments. The cellular activities were confirmed by MTT experiments using cancer cells. We employed computational studies to understand the intermolecular interactions at the atomic level. The identified chemotypes of **1**–**4** show limited similarities to the known inhibitors, but the core regions for hydrophilic interactions are shared. The detailed study of **1**–**4** will be helpful for developing new and potent Hsp90N inhibitors.

## Methods

### Selection of algorithm and best structure using the ensemble of experimental Hsp90 N-terminal domain structures

A search of the DALI database retrieved 108 structures of human Hsp90N, followed by alignment in the same direction to form an ensemble of structures^66^. Crystallographic waters were excluded. Ninety inhibitors found in Hsp90N crystal structures were docked into all the structures with five software programs, AutoDock^21^, AutoDock Vina^22^, DOCK 3.6^23^, DOCK 6.7^24^, and Glide^25,26^. Six algorithms, including two from DOCK 6.7 (anchor-and-grown and rigid), were applied with default parameters. To generate a dataset consisting of the known Hsp90N inhibitors and their physicochemically matched but topologically different decoys, the strongest 33 inhibitors were extracted from the BindingDB database based on IC_50_ or K_i_ values^27^. The DUD-E server then generated 2370 decoys^20^. An in-house written script, ALIS-DOCK (Automated pLatform for Integrative Structure-based DOCKing), automated SBVS by simultaneously using a Linux-cluster. The criteria used to choose the best structure for virtual screening were three metrics from the ROC curves [(area under the curve (AUC)^67^, logarithmically scaled AUC (LogAUC)^23^, and enrichment at 1% (EF1)^68^]. Once all molecules were arranged with their Glide scoring function in ascending order, the proportion of each molecule as false-positive or true-positive was plotted as *x* and *y* variables, respectively, to generate a ROC curve. The ROC curve was integrated to yield the AUC having a range of 0–1. Logarithmical scaling of the x-axis of the AUC from 0.001–1 expanded the range as −3–0, yielding a total area of 3. Once the value of AUC in the state was normalized by dividing by 3 and subtracting 0.145, the expected enrichment by chance, this was used to define the LogAUC. Therefore, the value of LogAUC lies in the range of −0.145 to 0.855. The values for AUC and LogAUC were expressed as percentages. The EF1 was defined as the enrichment factor in 1% of the compounds. For instance, EF1 of 10 indicates that 10% of the true-positives have lower scores than the molecule that is found in 1% of the false positives.

### High-throughput structure-based virtual screening and cheminformatics

Small molecules from the ChemBridge Express (San Diego, CA, U.S.A.) library were virtually screened using their corresponding molecules registered in ZINC^69,70^. The database supplied by the vendor was curated to contain only compounds satisfying the Lipinski rule of five, yielding approximately 450,000 compounds. The top 60 chemicals from two cases were chosen based on the scoring functions and purchased for assays. For the study of cheminformatics, the Tanimoto coefficient (Tc) was calculated based on Morgan circular fingerprint using RDKit (http://www.rdkit.org). The value of Tc lies between 0 and 1, where values closer to 1 indicate more chemical similarities.

### MD simulation

A series of MD simulations were performed in the apo and holo states using the AMBER 16 package supported by GPU^71,72^. Whereas Hsp90N was handled with the ff14SB force field, SQM and LEaP programs were used to prepare the GAFF force field for the inhibitors. The solvents, comprising TIP3P waters and Na and Cl ions for neutralization, solvated the apo or holo structures in an octahedron box. The thickness of the solvent shell from the protein was kept at least 10 Å. The Particle mesh Ewald (PME) method was used for long-range electrostatic interactions. Non-bonded interactions were truncated with 10 Å as a cut-off. All bonds involving hydrogen were constrained by the SHAKE method with an integration time step of 2 fs. Four stages comprised a run: minimization, heating, equilibrium, and production. Heating of the system from 0 to 300 K was done at a constant volume for 50 ps followed by 1500 cycles of minimization. For the next 100 ps, the equilibrium continued at a constant pressure of 1 atm and a temperature of 300 K. MD simulation for producing trajectories were followed for 102 ns using Langevin thermostat with a collision frequency of 2 ps^−1^ under a constant pressure of 1 atm. Analyses employed the later 100 ns with three runs starting from a structure with different random seeds. Trajectories were analysed using CPPTRAJ^73^.

### Purification of the Hsp90 N-terminal domain

The plasmid construct for overexpressing Hsp90N contains DNA corresponding to residues 1–235 of human Hsp90α. For stable isotope labelling, *Escherichia coli* strain BL21 (DE3) harbouring the expression plasmid was grown in M9 minimal medium and induced by 1 mM IPTG at 37 °C for 6 h. Harvested *E*. *coli* cells were lysed by sonication in 25 mM Tris buffer (pH 8.0) supplemented with 5 mM dithiothreitol (DTT), a tablet of Protease Inhibitor Cocktail (Roche), and 4 mM ethylene-diaminetetraacetic acid (EDTA). The soluble fraction of the cell lysate was applied to a 60 mL Sepharose Q FF column equilibrated with 25 mM Tris (pH 7.5), 2 mM EDTA, 2 mM DTT, and proteins were eluted with a linear gradient to 1 M NaCl. The protein was then concentrated using a Centriprep10 (Amicon) and purified by gel-filtration chromatography on a 350 mL Sephacryl S100 HR (2.6 × 65 cm) column in 20 mM Tris (pH 7.2), 0.1 M NaCl, 2 mM EDTA, and 2 mM DTT.

### NMR spectroscopy

All NMR spectra were recorded with Bruker Ascend 600 MHz spectrometer equipped with a TCI cryoprobe at 25 °C. Data were processed using TOPSPIN and were visualized using NMRView software^74^. The NMR buffer consisted of 50 mM phosphate buffer (pH 7.0) with 20 mM NaCl and 1 mM deuterated DTT, and 2.5% deuterated DMSO. To screen for small molecules that bind to Hsp90N, ^15^N-labelled protein and two small molecules were mixed with 1:1 stoichiometry at a concentration of 100 μM, leading to 30 data sets in total. In cases with significant changes in the signal of 2D [^1^H, ^15^N] HSQC spectra, additional HSQCs were recorded with an individual small molecule. For the backbone chemical shift assignments, standard triple resonance experiments were performed with ^13^C/^15^N-labelled Hsp90N. A series of 2D [^1^H, ^15^N] HSQC spectra were recorded by changing the ratios of the concentrations of the small molecule and protein to calculate dissociation constants (K_d_s) with the four hits using the TITAN (TITration ANalysis) package^32^. The chemical shifts of 2D [^1^H, ^15^N] HSQC in holo forms were assigned by tracking the peak changes and observing 3D HNCA of holo forms. 17-DMAG, a water-soluble geldanamycin analog, was used as a positive control.

### Differential scanning fluorimetry

DSF using a RT-PCR system (CFX connect, BioRad) assessed the stability of Hsp90N in the absence and presence of the hit compounds that were identified by 2D NMR^75^. Once Sypro-Orange (5×) was added into the solution of Hsp90N with each hit compound, the fluorescence from the dye was measured with excitation at 492 nm and emission at 610 nm in a temperature-dependent manner from 30 to 90 °C. The concentrations for Hsp90N and inhibitors were 2 and 200 μM, respectively. As a control, DMSO was mixed with the protein, and the fluorescence was measured with an identical procedure. The mid-point of the melting temperature, T_m_, was deduced by calculating the temperature that maximizes the differential curve of the profiles.

### Cell-based assays

Growth inhibition by the hit molecules were observed with MCF7, A549, and MCF10A cells, which are cell lines from breast cancer, lung cancer, and breast mammary gland, respectively. Cells were exposed to the hit compounds at concentrations ranging from 10 nM to 10 μM for 48 h, followed by a cell viability assay using 3-(4,5-dimethylthiazol-2-yl)-2,5-diphenyltetrazolium bromide (MTT). The final concentration of DMSO for each treatment was adjusted to be 0.1%. Each condition was repeated three times to measure uncertainty. The efficacy of each compound was compared to that of 17-DMAG. For reverse transcription and real-time PCR, MCF7 cells were treated with Hsp90 inhibitors, and then total RNA was isolated from the cells by using RNA iso plus (Takara) according to the manufacturer’s instructions. Reverse transcription of the 2-μg amount of RNA was performed by using M-MLV reverse transcriptase (Promega). Each real-time PCR reaction was performed in a 20-μl volume containing 100 ng of cDNA, 0.4 μM of each primer, and 2× the PowerUpTM SYBR Green Master Mix (Applied Biosystems). The primer sequences were as follows: human HSPA4, 5′-CTC GGC CAT GGA ATC TTT TA-3′ (forward) and 5′-CGC TAA TGA GTA TAG CGA CCG-3′ (forward); and 18S RNA, 5′-ACT CAA CAC GGG AAA CCT CA-3′ (forward) and 5′-AAC CAG ACA AAT CGC TCC AC-3′ (reverse). The real-time PCR reactions were run on a StepOnePlusTM Real-Time PCR Systems (Applied Biosystems). The comparative quantification of gene expression levels were normalized to the levels of the housekeeping gene (18S RNA). Relative gene expression changes are reported as number-fold changes compared to the control samples.

## Electronic supplementary material

Supplementary information
