# Supplementary material for: Structural ensemble-based docking simulation and biophysical studies discovered new inhibitors of Hsp90 N-terminal domain
Source: Sci Rep. 2018 Jan 10;8:368. doi: 10.1038/s41598-017-18332-8 (PMC5762686; doi:10.1038/s41598-017-18332-8)
Supplement: Supplementary file 1 — Supplementary information [file 41598_2017_18332_MOESM1_ESM.pdf]

Supplementary information

Structural ensemble-based docking simulation and biophysical studies discovered new inhibitors of Hsp90 N-terminal domain

Hyun-Hwi Kim[1,†], Ja-Shil Hyun[1,†], Joonhyeok Choi[2], Kwang-Eun Choi[2], Jun-Goo Jee[2,*] & Sung Jean Park[1,*]

From [1]College of Pharmacy and Gachon Institute of Pharmaceutical Sciences, Gachon University, Incheon 21936, Republic of Korea; [2]Research Institute of Pharmaceutical Researches, College of Pharmacy, Kyungpook National University, Daegu 41566, Republic of Korea

* Corresponding authors

Jun-Goo Jee, Ph.D.

College of Pharmacy, Kyungpook National University

80 Daehak-ro, Buk-gu, Daegu 41566, Republic of Korea

Phone, +82-53-950-8568; Fax, +82-53-950-8557

Email: jjee@knu.ac.kr

and

Sung Jean Park, Ph.D.

College of Pharmacy, Gachon University

191 Hambakmoero, Yeonsu-gu, Incheon 21936, Republic of Korea

Phone, +82-32-820-4957; Fax, +82-32-820-4829

Email: psjnmr@gachon.ac.kr

† These two authors contributed equally to this work.

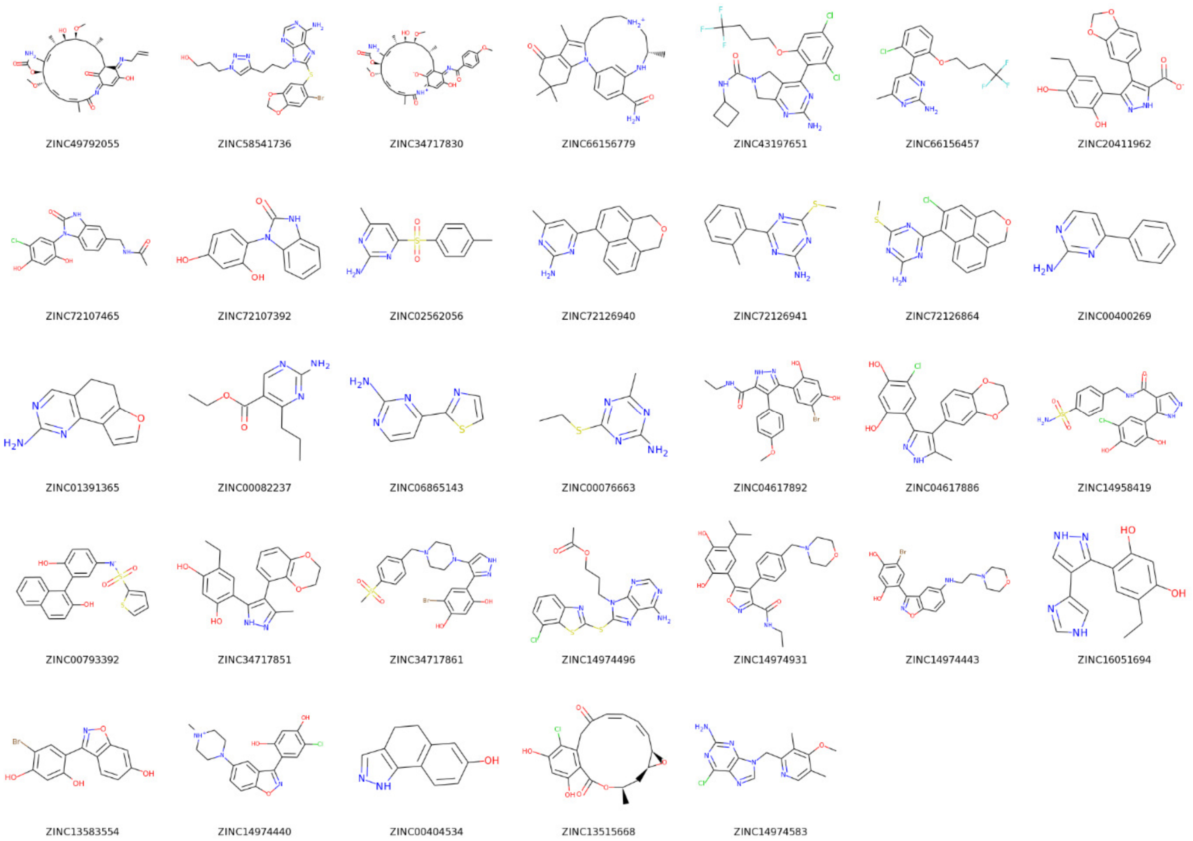

Figure S1. **The 33 inhibitors of Hsp90N used for generating decoys.** Corresponding ZINC IDs are shown.

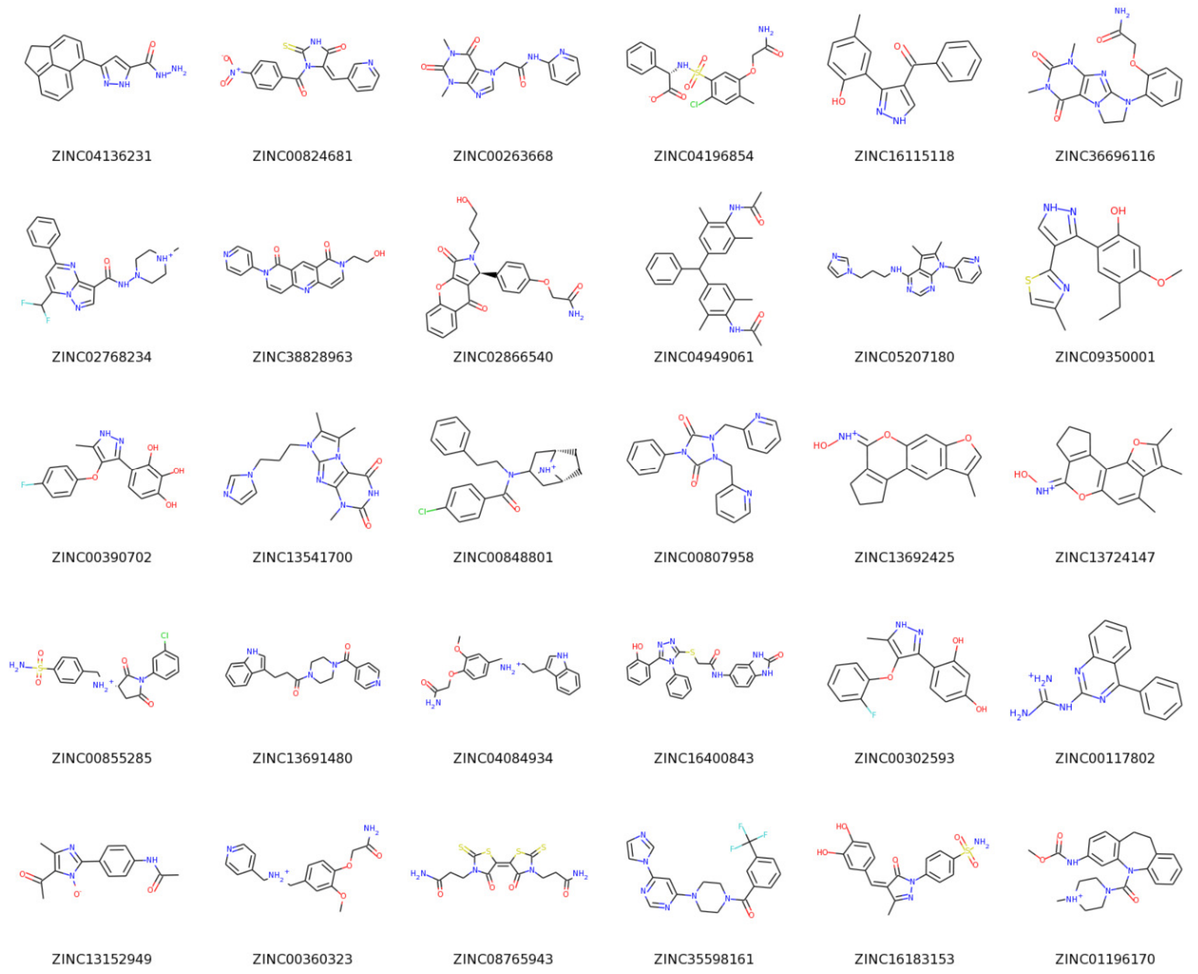

Figure S2. **The 60 purchased molecules for binding assays with Hsp90N.** The 30 molecules purchased for use in 2BYI-A. All the chemicals were ordered according to their Glide-SP scores. Two hits (**1** and **2**) are located 12th and 23rd.

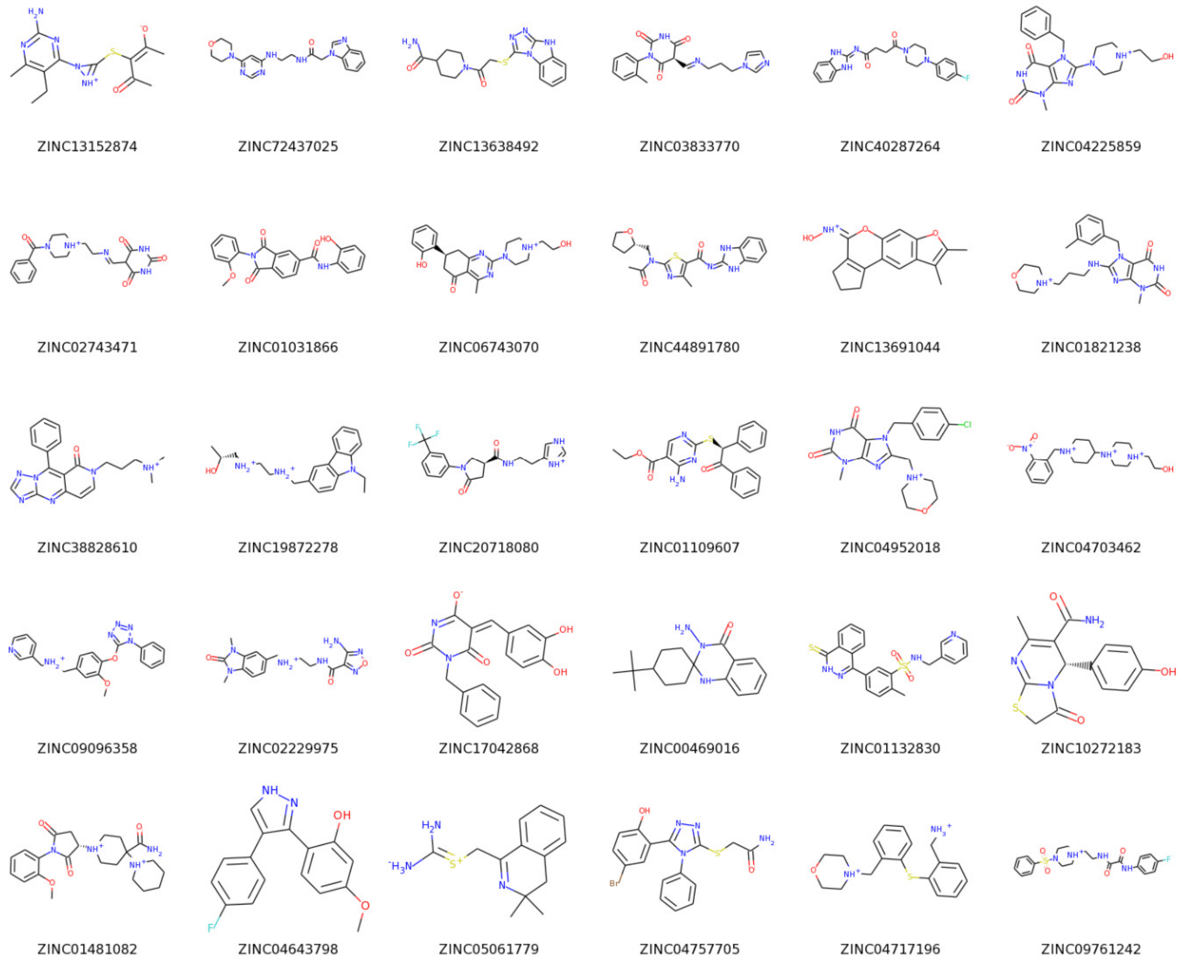

Figure S2. (continued) The 30 molecules purchased for use in 2YI5-A. Two hits (**3** and **4**) are located 26th and 28th.

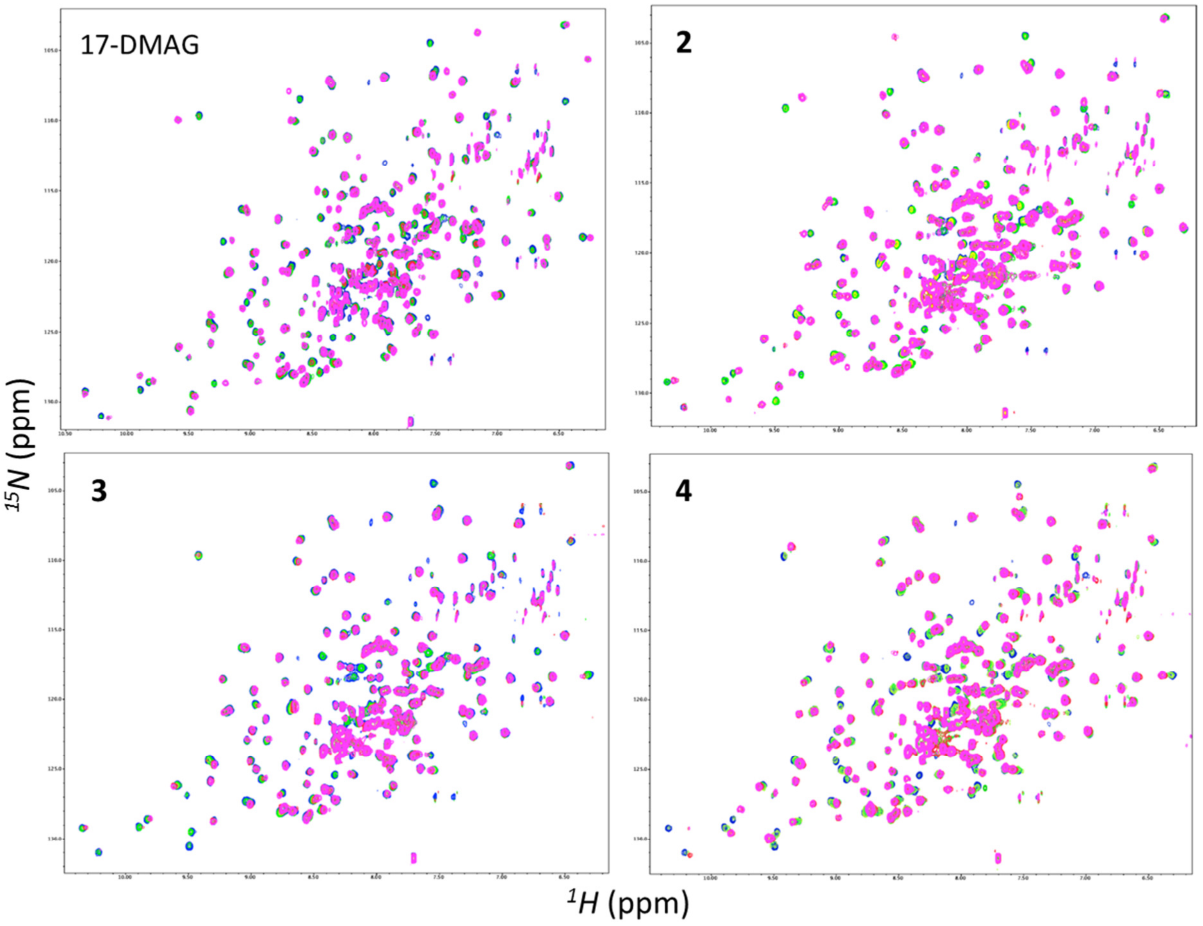

Figure S3. **Concentration-dependent changes in 2D [^{1}H, ^{15}N] HSQC spectra after addition of 17-DMAG and 2–4.** NMR peaks caused by a series of ligands with different concentrations in 1:0 to 1:2 protein:ligand ratios are shown. NMR peaks of 1:0 and 1:2 protein:ligand ratios are coloured in green and magenta, respectively.

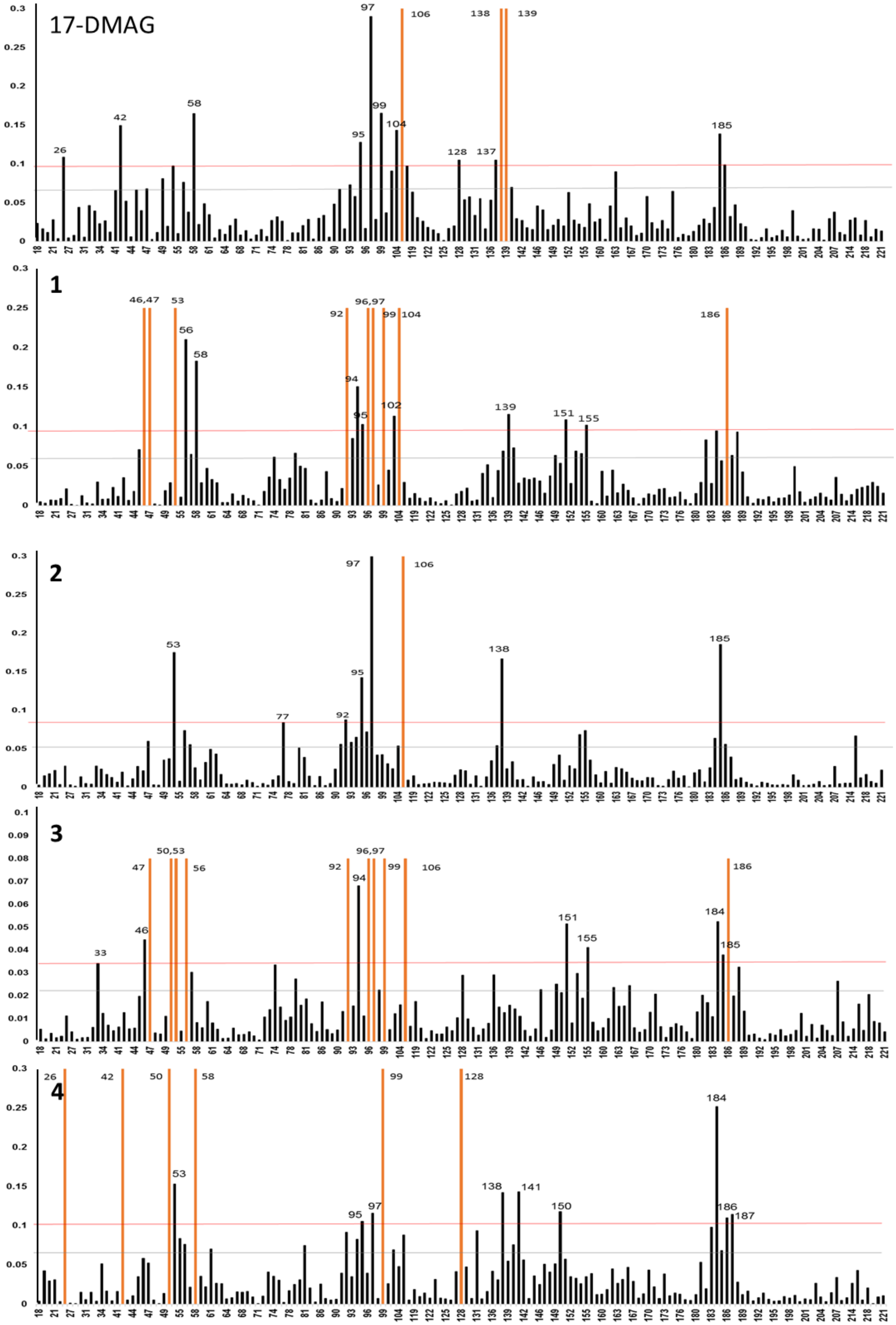

17-DMAG
97 106 138 139
58 42 26 99 104 95 128 137 185
1
46,47 53 96,97 92 99 104 186
56 58 94 95 102 139 151 155
2
97 106 53 95 138 185 77 92
3
50,53 96,97 47 56 92 94 99 106 186
46 33 151 155 184 185
4
26 42 50 58 99 128
184 53 138 141 150 95 97 186 187

Figure S4. **Residue-specific chemical shifts perturbation**. The x-axis and y-axis indicate the residue number and the perturbed chemical shifts (ΔCS) defined as $\sqrt{0.5 \times (\Delta H^2 + (0.2 \times \Delta N)^2)}$, respectively. Here ΔH and ΔN mean the difference of ^{1}H and ^{15}N chemical shifts between holo and apo states. The holo data were obtained in the condition of 1:2 protein:ligand ratio. Each inhibitor (17-DMAG and **1–4**) is written in the upper left corner. Once calculating the mean and standard deviation (SD) values of ΔCS in the cases of $\Delta CS > 0$, the residues of Hsp90N are classified into four criteria: (i) $\Delta CS \leq$ mean+SD, (ii) mean+SD < ΔCS ≤ mean+2SD, (iii) ΔCS > mean+2SD, and (iv) disappeared. Orange and grey horizontal lines indicate the values of mean+2SD and mean+SD, respectively. The residues with the disappeared peaks are represented with orange vertical bar.

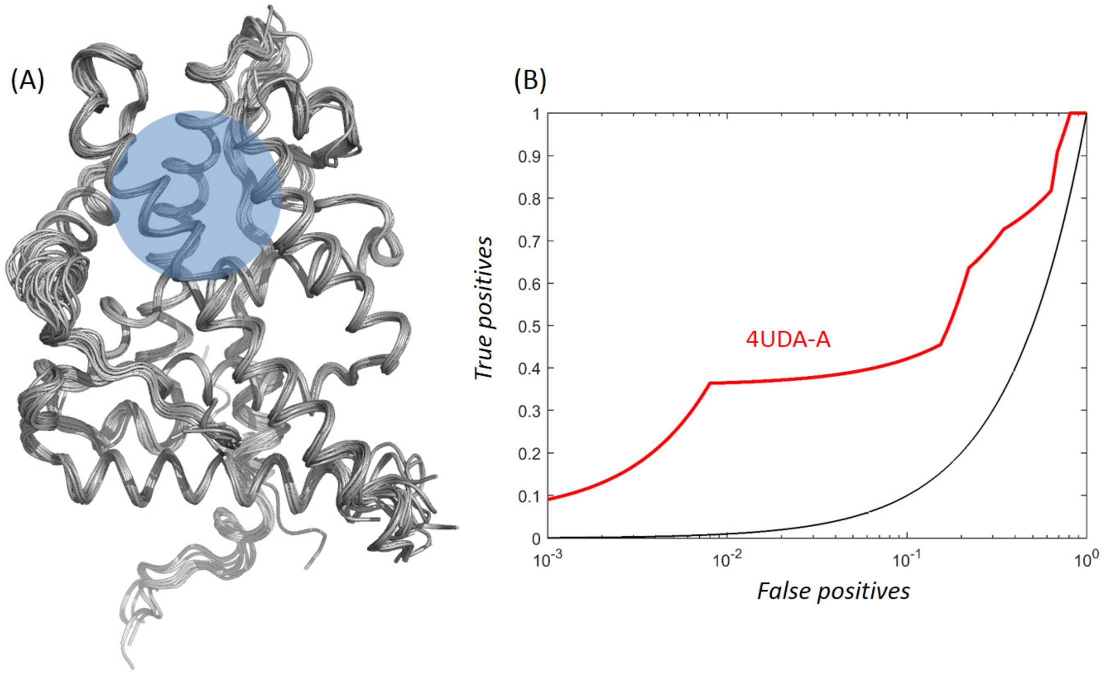

Figure S5. **Structural ensemble and structure-dependent profile of ROC curve in mineralocorticoid receptor.** (A) The ensemble of 36 crystal structures of mineralcorticoid receptor is represented with the inhibitor-binding pocket coloured in blue. (B) Receiver operating characteristic (ROC) curve from the structure of 4UDA-A is drawn in red. Random enrichment is shown in black for comparison. In the ROC curves, the x-axis is logarithmically scaled to emphasize the earlier enrichments of true-positives. The values of AUC, LogAUC, and EF1 for 4UDA-A are 75.7, 29.0, and 34.8, respectively.

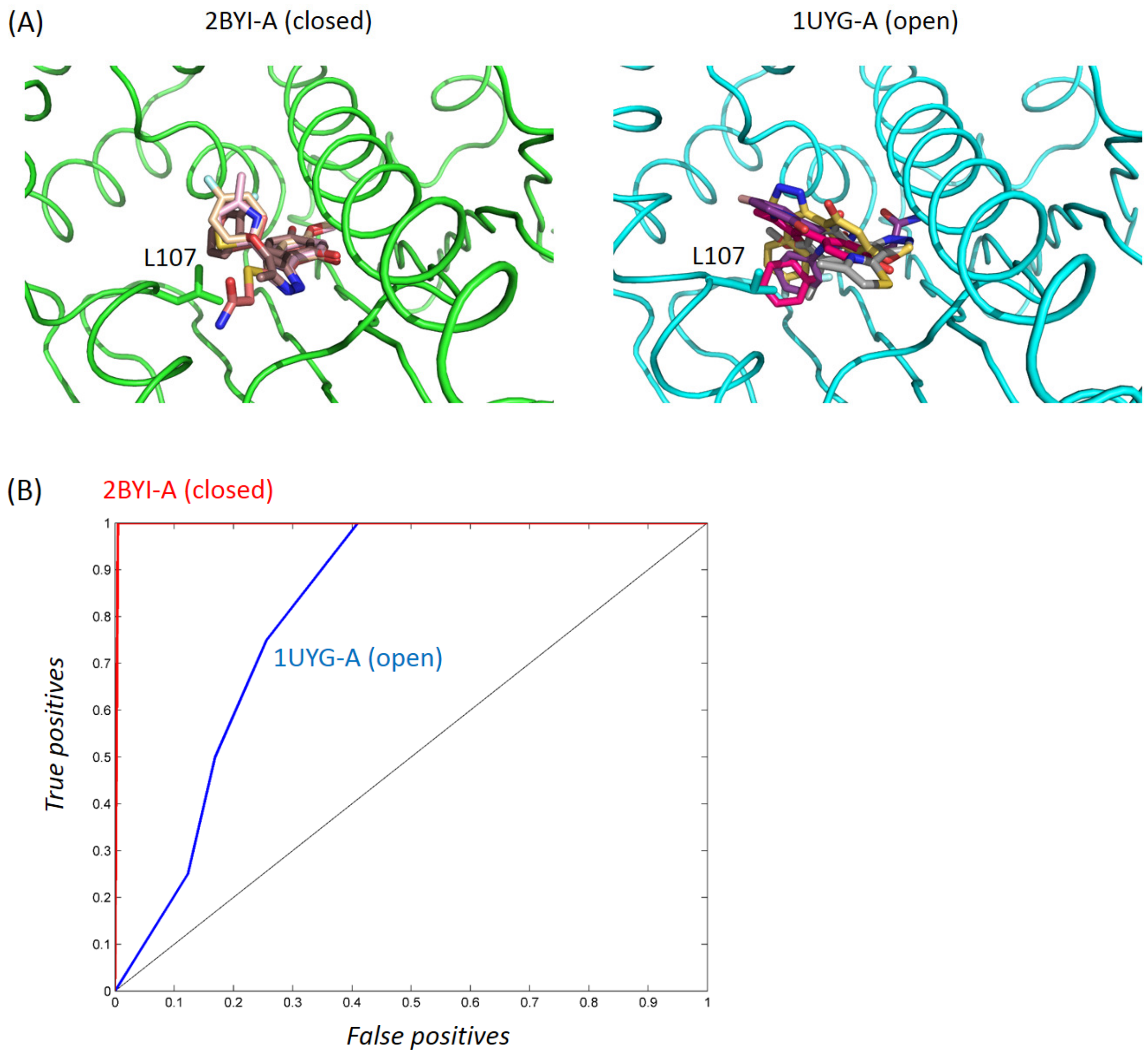

Figure S6. **Overlaid docked poses and enrichments of 1–4 in the closed and open forms of Hsp90N.** (A) **1–4** were docked into 2BYI-A and 1UYG-A for the closed and open forms, respectively. The positions of Leu-107 were drawn. (B) Enrichments of **1–4** were shown using receiver operating characteristic curves (ROCs). DUD-E server generated property-matched 200 decoys based on **1–4**. Docking with both **1–4** and decoys followed. Area under the curves with ROCs of 2BYI-A and 1UYG-A were 99 and 81%, respectively.
